# Fluoroquinolones increase the risk of aortic aneurysm and dissection: A protocol for meta-analysis

**DOI:** 10.1097/MD.0000000000028081

**Published:** 2021-12-23

**Authors:** Jiawei Zhang, Zhe Zhang

**Affiliations:** a Department of Vascular Surgery, The First Hospital of China Medical University, Liaoning, China; b Operating Room, The First Hospital of China Medical University, Liaoning, China.

**Keywords:** aortic aneurysm, aortic dissection, fluoroquinolones, meta-analysis

## Abstract

**Background:**

Fluoroquinolones have been associated with collagen degradation, raising safety concerns related to more serious collagen disorders with use of these antibiotics, including aortic aneurysm and dissection. We performed this protocol for meta-analysis to examine the relationship between fluoroquinolone therapy and the risk of developing aortic aneurysm and dissection.

**Methods:**

This study will be designed following the guidelines of the Preferred Reporting Items for Systematic Reviews and Meta-Analyses Protocols statement guidelines. Studies were identified through systematic searches in November 2021 with no restrictions on date and time, and publication status using the following bibliographic databases: Embase, Medline, PubMed, Web of Science, Science Direct, and the Cochrane Library. The risk of bias of included studies were estimated by taking into consideration the characteristics including random sequence generation, allocation concealment, blinding of patients, blinding of outcome assessment, completeness of outcome data, selective reporting and other bias by Cochrane Collaboration's tool. Data synthesis and analyses were performed using Stata version 10.0 software.

**Results:**

The results of this systematic review and meta-analysis will be published in a peer-reviewed journal.

**Conclusion:**

Use of fluoroquinolones may be associated with an increased risk of aortic aneurysm and dissection. While these were rare events, physicians should be aware of this possible drug safety risk associated with fluoroquinolone therapy.

**Open Science Framework registration number:**

https://doi.org/10.17605/OSF.IO/ZKE3Y10.17605/OSF.IO/UP3BA

## 1 Introduction

Aortic aneurysm and aortic dissection are rare events. Aortic aneurysm is defined as a permanent localized dilation of the aorta resulting in at least a 50% increase in the aortic diameter.^[1]^ Most patients are asymptomatic until they experience a complication (aortic aneurysm rupture). Unless treated immediately, aortic aneurysm rupture leads to massive internal bleeding. Aortic dissection is the development of a tear in the aortic intima that creates a false lumen through the aortic media.^[2]^

The reported incidence for aortic dissection is 2.9/100,000 case/patient/year.^[3]^ The prevalence of abdominal aortic aneurysms is reported to be up to 8% in men older than 65 years.^[4]^ Despite their low occurrence, aortic aneurysm and aortic dissection count as the most severe collagen-related adverse events because they can lead to life-threatening conditions like rupture of an aortic aneurysm or acute aortic dissection.^[5,6]^ The known risk factors for aortic aneurysm and aortic dissection include congenital connective tissue disorders, older age, male sex, atherosclerotic cardiovascular disease, and tobacco smoking.^[7,8]^

The fluoroquinolones class of antibiotics (e.g., ciprofloxacin, levofloxacin, moxifloxacin) has been used to treat a broad range of infections ranging from urinary tract infections to respiratory infections.^[9]^ Several observational trials have shown an association between the use of fluoroquinolones and the development of collagen-related adverse events including tendon rupture, retinal detachment, aortic aneurysm, or aortic dissection.^[10–13]^ This was thought to be linked, in part to fluoroquinolone-related oxidative stress resulting in degenerative changes to the extracellular matrix components.

Given the high risk of a fatal outcome from aortic aneurysm or dissection and the widespread use of fluoroquinolones, exploring the association between fluoroquinolones and aortic aneurysm or dissection is of particular clinical interest. Therefore, we performed a protocol for meta-analysis to explore the risk of an aortic aneurysm or aortic dissection following fluoroquinolone administration.

## 2 Methods

This meta-analysis was registered at Open Science Framework registries (registration number: 10.17605/OSF.IO/UP3BA https://doi.org/10.17605/OSF.IO/2CN5R) and was conducted in accordance with the guidelines of the Preferred Reporting Items for Systematic Reviews and Meta-Analyses Protocols statement guidelines.^[14]^ Ethics application was not required as this study is based on published trials.

### 2.1 Search strategy

Studies were identified through systematic searches in November 2021 with no restrictions on date and time, and publication status using the following bibliographic databases: Embase, Medline, PubMed, Web of Science, Science Direct, and the Cochrane Library. The keywords used in the search strategy were: fluoroquinolone, besifloxacin, ciprofloxacin, enoxacin, gatifloxacin, levofloxacin, moxifloxacin, norfloxacin, ofloxacin, pefloxacin, aortic aneurysm, and aortic dissection. Boolean operators such as “AND” and “OR” were used to combine search terms. The reference lists of the included studies were also checked for additional studies that were not identified with the database search.

### 2.2 Eligibility criteria

We included randomized controlled studies that had fluoroquinolones as intervention and/or exposure group and a control group. The fluoroquinolones group included individuals treated and/or exposed to any fluoroquinolones, with any route of administration, dose, treatment duration or indication, without restriction of age. The control group was composed by individuals receiving placebo, no treatment, absence of exposure to fluoroquinolones, or exposed to non-fluoroquinolones antibiotics.

### 2.3 Data extraction

Two independent authors will extract the below descriptive information from the included articles: demographic information of patients, such as average age, number of patients, sex ratio and body mass index; study characteristics, such as authors, year of publication, study language, study design, and the average follow-up period; details of interventions and outcome measures. If the data cannot be directly extracted or is missing, we will contact the relevant author to ensure that the information is complete. Otherwise, we will calculate them with the guideline of Cochrane Handbook for Systematic Reviews of Interventions 5.1.0.

### 2.4 Quality evaluation

The risk of bias assessment of the included studies was performed by 2 authors independently using the Cochrane Collaborations risk of bias tool. This tool included 7 aspects which were sequence generation (selection bias), allocation sequence concealment (selection bias), blinding of participants and personnel (performance bias), blinding of outcome assessment (detection bias), incomplete outcome data (attrition bias), selective outcome reporting (reporting bias), and other bias (baseline balance and fund). Additionally, each of the aspects was ranked low risk of bias, high risk of bias, and unclear risk of bias.

### 2.5 Statistical analysis

We performed the meta-analysis by Stata version 10.0 software and calculated the statistics using the inverse variance statistical method. Continuous variables were expressed as the weighted mean difference or standardized mean difference and 95% confidence interval (CI). Weighted mean difference was used when data were measured in the same scale and standardized mean difference were used if data were measured using different scales. Heterogeneity among the studies was quantified with the *I*^2^ statistic. If *I*^2^ > 50% or *P* < .1, a random effect model was used to decrease heterogeneity, and the subgroup and sensitivity analysis were performed to explore the sources of heterogeneity; otherwise, heterogeneity was negligible and a fixed-effect model was used. To evaluate publication bias, we perform a funnel plot if the number of included studies is sufficient (>10 articles). A symmetrical funnel plot indicates no possibility of publication bias, while an asymmetrical funnel plot indicates a high possibility of publication bias. If we identify publication bias through analysis of the funnel plot, we may discuss possible reasons such as small-study effects.

## 3 Discussion

While the exact mechanism of how use of fluoroquinolones can cause aortic aneurysm and dissection is unknown, there are several possibilities. The strength of the aortic wall relies on the structural integrity of the extracellular matrix proteins, which are regulated by proteolytic enzymes such as matrix metalloproteinases (MMPs).^[15]^ It has been demonstrated that MMPs play an important role in the pathogenesis of aortic aneurysm and dissection. Dysregulation of MMP production and activity leads to extracellular matrix degradation and medial layer degeneration.^[16]^ Examination of smooth muscle cells from abdominal aortic aneurysm shows an upregulated expression of MMP-9 and MMP-2.^[17]^ Fluoroquinolones have been shown to affect the synthesis of collagen in the tendon cell and disorganization of the extracellular matrix in the cornea. Animal studies have shown that ciprofloxacin may induce the expression of MMP-9 and MMP-2 in cornea tissue and MMP-2 in tendon tissue.^[18]^ Both MMP-2 and MMP-9 are gelatinases that have collagenolytic activity. Collagen and elastin are the primary extracellular matrix components of the aortic wall, and these elements make up approximately 50% of the dry weight of normal arteries. Therefore, it is possible that fluoroquinolones destroy the collagen and connective tissue along the aortic wall causing aortic aneurysm and dissection as they do on tendon and cornea. This protocol may provide evidence regarding the association of fluoroquinolones with the risk of aortic aneurysm or aortic dissection.

## Author contributions

**Conceptualization:** Zhe Zhang, Jiawei Zhang.

**Data curation:** Zhe Zhang.

**Funding acquisition:** Zhe Zhang.

**Investigation:** Zhe Zhang, Jiawei Zhang.

**Methodology:** Zhe Zhang.

**Writing – original draft:** Jiawei Zhang.

**Writing – review & editing:** Zhe Zhang.
